# TOR signalling mediates the collective movement of border cells in *Drosophila* oogenesis

**DOI:** 10.1242/dev.204612

**Published:** 2025-09-04

**Authors:** Sudipta Halder, Adhisree Sharma, Sayan Acharjee, Neha Biju, Mincy Kunjumon, Rohan Jayant Khadilkar, Mohit Prasad

**Affiliations:** ^1^ Department of Biological Sciences, Indian Institute of Science Education & Research- Kolkata, Mohanpur Campus, Mohanpur - 741246, Nadia, West Bengal, India; ^2^ Department of Biomedical Genetics, University of Rochester Medical Center, Rochester, New York, NY 14642, USA; ^3^ Stem cell and Tissue Homeostasis lab, CRI, ACTREC – Tata Memorial Centre, Kharghar, Navi Mumbai 410210; ^4^ Homi Bhabha National Institute, Anushaktinagar, Mumbai 400094, India

**Keywords:** TOR signalling, REPTOR, Diap1, BC migration, *Drosophila* oogenesis, Collective cell movement, BIRC2

## Abstract

Collective cell migration is seen in various biological processes spanning embryonic development, organogenesis, wound healing and, unfortunately, cancer metastasis. Here, we have examined the role of the evolutionarily conserved Target of Rapamycin signalling (TOR) in mediating collective cell movement employing the model of migrating border cells (BCs) in *Drosophila* oogenesis. Although TOR signalling is classically linked to cell growth, cell proliferation and metabolism, here we demonstrate that TOR complex 1 (TORC1) regulates efficient group cell movement of BCs. Employing live cell imaging, genetics, and tissue immunohistochemistry, we demonstrate that TOR functions through the transcription factor REPTOR to modulate Death-associated inhibitor of apoptosis 1 (Diap1) in mediating efficient movement of BCs. Coincidentally, rapamycin-treated myeloblast Kasumi-1 cells exhibit lower levels of transcript for the Diap1 homologue baculoviral IAP repeat-containing 2 (BIRC2), similar to what is observed in flies.

## Teaser

**Summary:** TORC1 functions through Diap1 to mediate the collective movement of BCs in *Drosophila* oogenesis, an axis of regulation that is also conserved in leukaemic cells.

## INTRODUCTION

In metazoans, collective movement is integral to various cellular processes spanning early development, organ formation, tissue regeneration and tumour migration. Collectively migrating cells are polarised and they acquire diverse constellations, either as sheets or small cohorts, as a result of different adhesive properties. Although widespread, the underlying mechanism of multicellular cell movement is far less understood compared to that of single cells.

We specifically focused on Target of Rapamycin (TOR) signalling as it has been linked to cell transformation and progression of various human cancers (Panwar et al., 2023; Pópulo et al., 2012). TOR is known to couple nutrient availability with cellular growth and is highly conserved from yeast to mammals (González and Hall, 2017). In response to different signalling inputs, TOR functions through two multi-subunit protein complexes, TOR complex 1 (TORC1) or TOR complex 2 (TORC2), to modulate diverse cellular processes associated with growth, proliferation, survival and autophagy. Although the components of TOR are well characterised, we have gained insights into its diverse targets and how it facilitates various processes beyond its classical role. Any event that hyperactivates TOR stimulates protein synthesis, favours cell growth and aids evasion of apoptosis (Loewith and Hall, 2011; Wullschleger et al., 2006). Studies on cells in culture have demonstrated that TORC1 modulates cell movement primarily through post-translational events that assemble focal adhesions, stimulate actin polymerisation, and facilitate tissue remodelling (Liu and Parent, 2011; Liu et al., 2008). Depending on the cell types, TORC2 can regulate the actin cytoskeleton and cell polarity. Thus, it is clear that TOR can modulate cell movement. All our current understanding in this context is mainly based on *in vitro* studies carried out on cells in culture. It remains to be determined how these findings will extrapolate to *in vivo* settings, specifically in multicellular contexts whereby a group of cells moves as a cohort and responds collectively to diverse extracellular signals.

Over the years, border cell (BC) migration in *Drosophila* oogenesis has emerged as a model for studying group cell movement. The fly ovary consists of several oval structures called egg chambers that go through 14 stages of development to produce mature eggs. Each egg chamber consists of 16 centrally located germ cells enveloped by a layer of cuboidal epithelial cells of somatic origin called follicle cells. Their ends are marked by the presence of a pair of specialised follicle cells called the polar cells. In response to a cytokine secreted from the anterior polar cells, a group of six to eight anterior follicle cells activates the C/EBP transcription factor slow border cells (Slbo), through STAT (Stat92E) and acquires a migratory fate. The cells then detach and move towards the oocyte. This migrating cluster is referred to as the BCs (Montell et al., 1992; Silver and Montell, 2001). It has been demonstrated that TORC1 negatively regulates STAT in follicle cells, influencing BC fate. Under TOR-depleted conditions, larger BC clusters are formed, exhibiting a delay in forward movement. Although STAT downregulation in TOR-depleted follicle cells suppresses the excessive BC fate, intriguingly it failed to rescue the BC migration defect (MD) (Kang et al., 2018). This suggests that TOR may have a distinct role in mediating BC migration that is independent of its effect on their fate specification.

Coupling genetics, live-cell imaging and immunohistochemistry, we demonstrate that TOR mediates the efficient movement of BCs. We demonstrate that TOR maintains F-actin in the migrating cluster. As overexpression of Diap1 rescues the MD of the TOR-depleted BCs, we propose that Diap1 is a target of TOR in mediating collective cell movement. Strikingly, we observe that the mammalian counterpart of the TORC1-Diap1 (BIRC2) axis is conserved in myeloblast-derived Kasumi-1 cell lines. Altogether, our results provide molecular insight into how TOR signalling modulates collective cell migration.

## RESULTS

### TOR is required for BC migration

To test our hypothesis, we generated TOR (mTor) knockout clones using a *TOR*^Δ*P*^ null allele, using the mosaic analysis with a repressible cell marker (MARCM) technique (Wu and Luo, 2007; Zhang et al., 2000). Notably, we observed a BC MD in 60.6% of egg chambers carrying *FRT 40A-TOR*^Δ*P*^ mutant clones (*n*=61), whereas no MD was observed in 98 egg chambers that carried control *FRT 40A* BC clones (Fig. S1A-E). As TOR is known to modulate BC fate, we evaluated the number of cells in the migration-defective clusters. We found that the *TOR*^Δ*P*^ mutant BC clusters exhibiting MD had more cells compared to those observed in the controls (9.0±0.18 cells in *TOR*^Δ*P*^ egg chambers versus 6.3±0.36 cells in *FRT 40A* egg chambers) (Fig. S1F-H). Although our results recapitulate the previous findings of Kang et al. (2018) that TOR depletion affects both BC fate and their efficient movement, a caveat of the MARCM approach is that mutants are generated early, which affects both BC fate and their migration. Thus, using this method, one cannot study the role of TOR solely in migration, independently of its effect on BC fate specification.

**Fig. 1.**
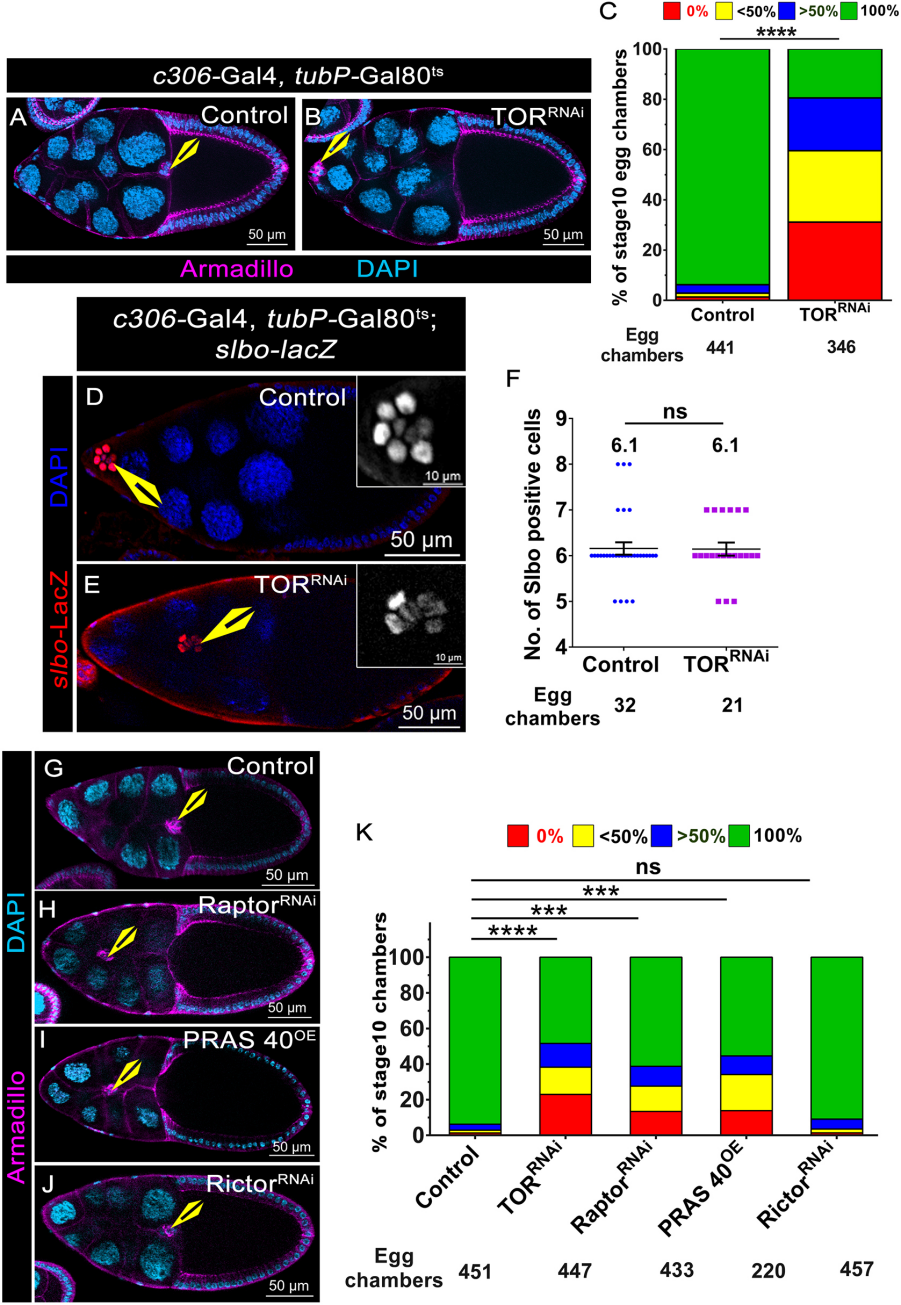
***Drosophila* TOR regulates BC migration during oogenesis.** (A,B,G-J) Stage 10 egg chambers of the indicated genotype; Armadillo (magenta) and DAPI (cyan). Yellow arrowheads indicate BCs. (C) Quantification of migration efficiency for the egg chambers shown in A,B.; ****P<0.0001 (non-parametric Mann–Whitney test). *N*≥3. The colour code indicates the distance covered from the anterior end. (D,E) Stage 9 egg chambers of the indicated genotype; *slbo-lacZ* (red; inset, grey) and DAPI (blue). Yellow arrowheads indicate BCs. (F) Quantification of the number of Slbo-positive cells. Error bars indicate s.e.m. ns, not significant (*P*≥0.05). *N*=3. Yellow arrowheads indicate BCs. (K) Quantification of migration efficiency for the egg chambers shown in G-J. The colour code indicates the distance covered from the anterior follicle cell; ****P<0.0001, ***P<0.001 (non- parametric Mann–Whitney test). ns, not significant (*P*≥0.5). *N*≥3. biological replicates of individual experiment.

To circumvent this problem, we employed the RNA interference (RNAi) approach to downregulate TOR function in the BCs after they had been specified. This would allow us to study its role specifically in migration without affecting their fate. We overexpressed TOR^RNAi^ under temperature-restrictive conditions using *tubP*-Gal80^ts^ and observed that 80% of TOR-depleted BC clusters failed to complete migration compared to only 5% observed in the controls (Fig. 1A-C) (McGuire et al., 2003). Notably, the number of BCs in the TOR^RNAi^-overexpressing cluster was similar to that of the control (*n*>20 egg chambers), suggesting that this method of TOR downregulation did not affect BC fate (Fig. 1D-F). We also noted that 25% of the TOR-depleted BC clusters failed to detach from the anterior follicle cells compared to 1% in the control egg chambers (Fig. 1C). In addition, polar cell-specific TOR depletion did not impede BC migration, suggesting that TOR plays a significant role in the outer BCs to modulate the cluster movement (TOR^RNAi^, 2.34%; control, 2.63%) (Fig. S2A-C). Altogether, these results suggest that the TOR function is required in the outer BCs for cluster movement.

Next, we were curious to determine how TOR was mediating BC movement.

### BC migration is dependent on TORC1

The TOR pathway is mediated through two TOR complexes: TORC1 and TORC2. These complexes modulate several downstream targets that ultimately regulate diverse physiological processes within a cell. TORC1 is characterised by the presence of the macromolecular adaptor protein Raptor, which regulates cell growth, metabolism and autophagy (Liao et al., 2008). Rictor forms an essential part of TORC2 that phosphorylates downstream targets and modulates cellular activities linked to cytoskeletal rearrangement, tissue growth, stress responses and control of mitochondrial quality. We altered the function of each complex and checked its effect on BC movement.

To investigate TORC1 function, we independently manipulated Raptor and Proline-rich Akt substrate 40 kDa (PRAS40) in migrating BCs. Similar to TOR^RNAi^ (51% MD), we observed that 38.75% of Raptor^RNAi^-overexpressing BC clusters exhibited a MD (Fig. 1H). PRAS40 is an inhibitor of TORC1, and, as expected, its overexpression impeded BC movement in 44% of egg chambers (Fig. 1G,I,K). Overexpression of Raptor^RNAi^ and PRAS40 in the follicle cells resulted in lower levels of phosphorylated Ribosomal protein S6 (pS6), a readout of TORC1, suggesting that they are indeed effective and downregulate TORC1 (Fig. S3A-B1). By contrast, overexpression of Rictor^RNAi^ to deplete TORC2 function resulted in only 9% of egg chambers exhibiting MD compared to 6% in the control (Fig. 1J,K, Fig. S3E-I). RT-PCR analysis indicated that the Rictor^RNAi^ lines employed were effective, exhibiting more than 50% downregulation at the transcript level (Fig. S3C,D). Altogether, our analysis suggests that depleting the function of TORC1 impedes BC movement. Since Rictor^RNAi^ did not affect BC movement significantly, we believe that TORC1 plays a more dominant role in BC cluster movement than TORC2.

### TOR-depleted BC clusters migrate slower

**Fig. 2.**
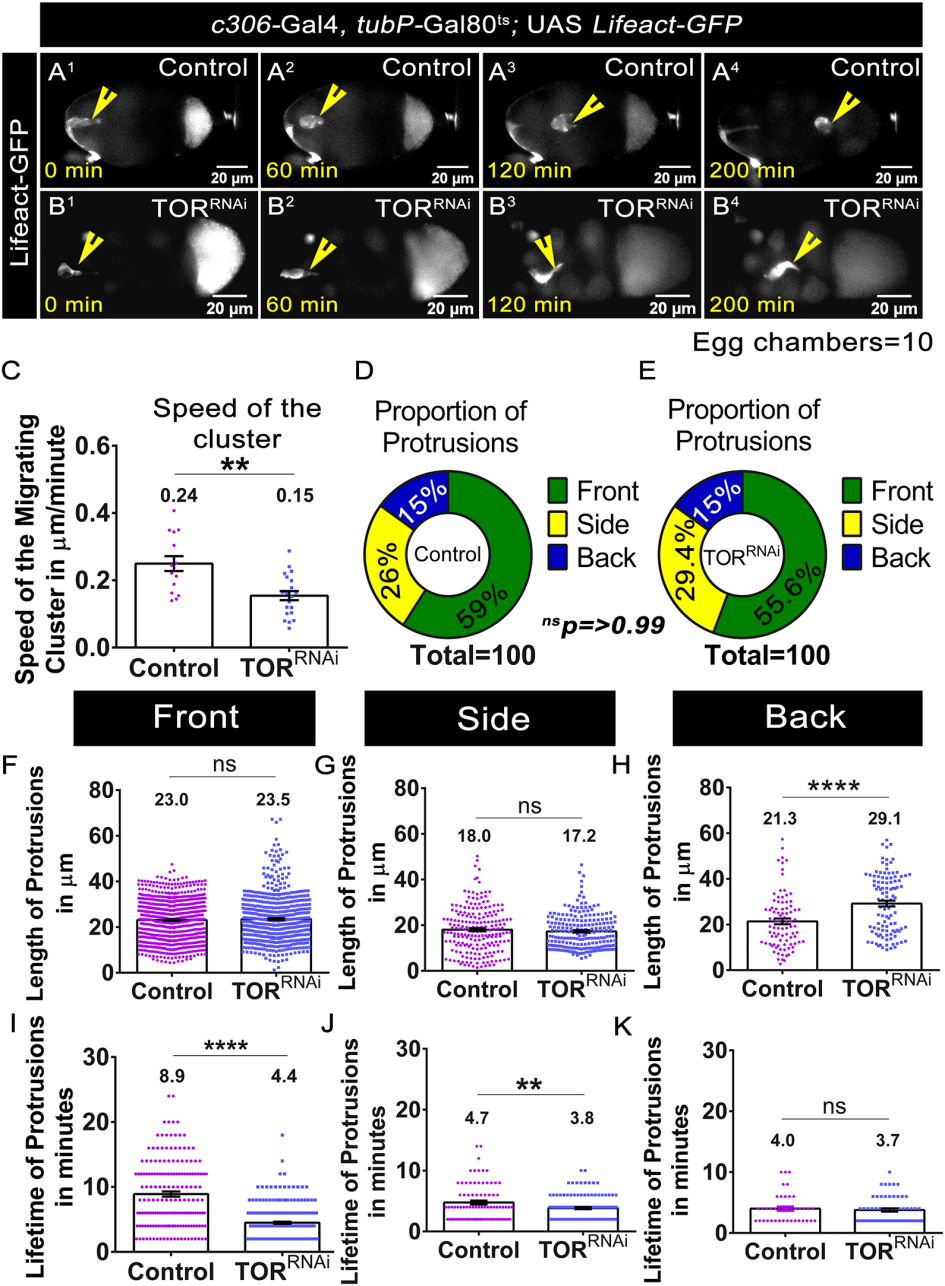
**TOR regulates protrusion dynamics in migrating clusters.** (A1-B4) Time-lapse snapshots of stage 9 egg chambers of the indicated genotypes. Yellow arrowheads mark BC clusters. Note that TOR^RNAi^ clusters exhibit a migration delay. (C) Comparison of migration speed between control and TOR-depleted clusters. (D,E) Radial diagram depicting protrusion distribution in a two-dimensional plane. (F-K) Effect of TOR^RNAi^ on the protrusion length (F-H) and protrusion stability (I-K). All error bars represent s.e.m. **P<0.01, ****P<0.0001 (non-parametric Mann–Whitney test). ns, not significant (*P*≥0.05). *n*=10 egg chambers.

To understand how TOR depletion impedes the movement of BCs, we performed time-lapse imaging of migrating clusters (Prasad et al., 2007). For live imaging, we used *c306*-Gal4 to drive UAS *Lifeact-GFP*, enabling us to visualise BC clusters and study the protrusive and retractive behaviour of clusters in real time (Riedl et al., 2008). Analysis of time-lapse movies revealed that TOR depletion impedes BC migration (Fig. 2A1-B4, Movies 1 and 2). The speed analysis was performed only for the detached BC clusters during their active phase of migration. We observed that TOR-depleted clusters (average speed of 0.15±0.1 μm/min, *n*=21 movies) migrated slower compared to the controls (0.24±0.1 μm/min, *n*=16 movies) (Fig. 2C). As the TOR-depleted clusters were slower, we analysed the protrusive behaviour of these clusters. Unexpectedly, we did not observe any significant difference in the distribution of the protrusions being extended between the control and the TOR-depleted clusters (Fig. 2D,E). To pick up subtle differences, we categorised the direction, length and stability of the protrusion from the migrating cluster. The major fraction of the protrusions from the control cluster were directed towards the oocyte (forward), and very few were directed sideways or rearward towards the anterior follicle cells (Felix et al., 2015). The average length of frontal and sidewards protrusions from TOR-depleted clusters was not significantly different from those of the control (Fig. 2F,G). However, the TOR-depleted clusters exhibited longer rearward protrusions (control, 21.3±1.2 μm; TOR^RNAi^, 29.1±1.2 μm) (Fig. 2H). When we analysed the stability of protrusions, we found that the lifetime of forward and sideward protrusions was significantly decreased in TOR-depleted clusters compared to the controls (average protrusions lifetime: front, 8.9±0.39 min in control versus 4.4±0.15 min in TOR^RNAi^; side, 4.7±0.33 min in control versus 3.84±0.17 min in TOR^RNAi^; back, 4.0±0.36 min in control versus 3.7±0.26 min in TOR^RNAi^) (Fig. 2I-K; ten movies each). In summary, real-time analysis of migrating BCs revealed that TOR is required for the generation of stable, forward-directed protrusions and suppresses rearward protrusions, thereby facilitating the efficient movement of clusters (Movies 1-3).

It is known that F-actin is associated with the dynamics of cellular protrusions (Le Clainche and Carlier, 2008). Since we observed altered protrusion dynamics in TOR-depleted clusters, we stained the BC cluster with rhodamine phalloidin to check the status of F-actin. We observed that the mean intensity of rhodamine phalloidin was lower in TOR-depleted BC clusters compared to the control (average intensity in control, 1.7; in TOR^RNAi^, 0.6; Fig. S4A1-C). We also observed that the TOR-depleted clusters exhibited a smaller number of F-actin fibres compared to the control (average number of F-actin fibres in control, 55.2; in TOR^RNAi^, 28.8; Fig. S4A1-B2,D). Altogether, our results suggest that the downregulation of TOR affects protrusions and F-actin in migrating BCs, which may impact their migration efficiency.

As TORC1 is known to mediate both transcription and translation of downstream targets genes, we next set out to determine how it was affecting the BC movement.

### BC migration is negatively regulated by REPTOR activity

**Fig. 3.**
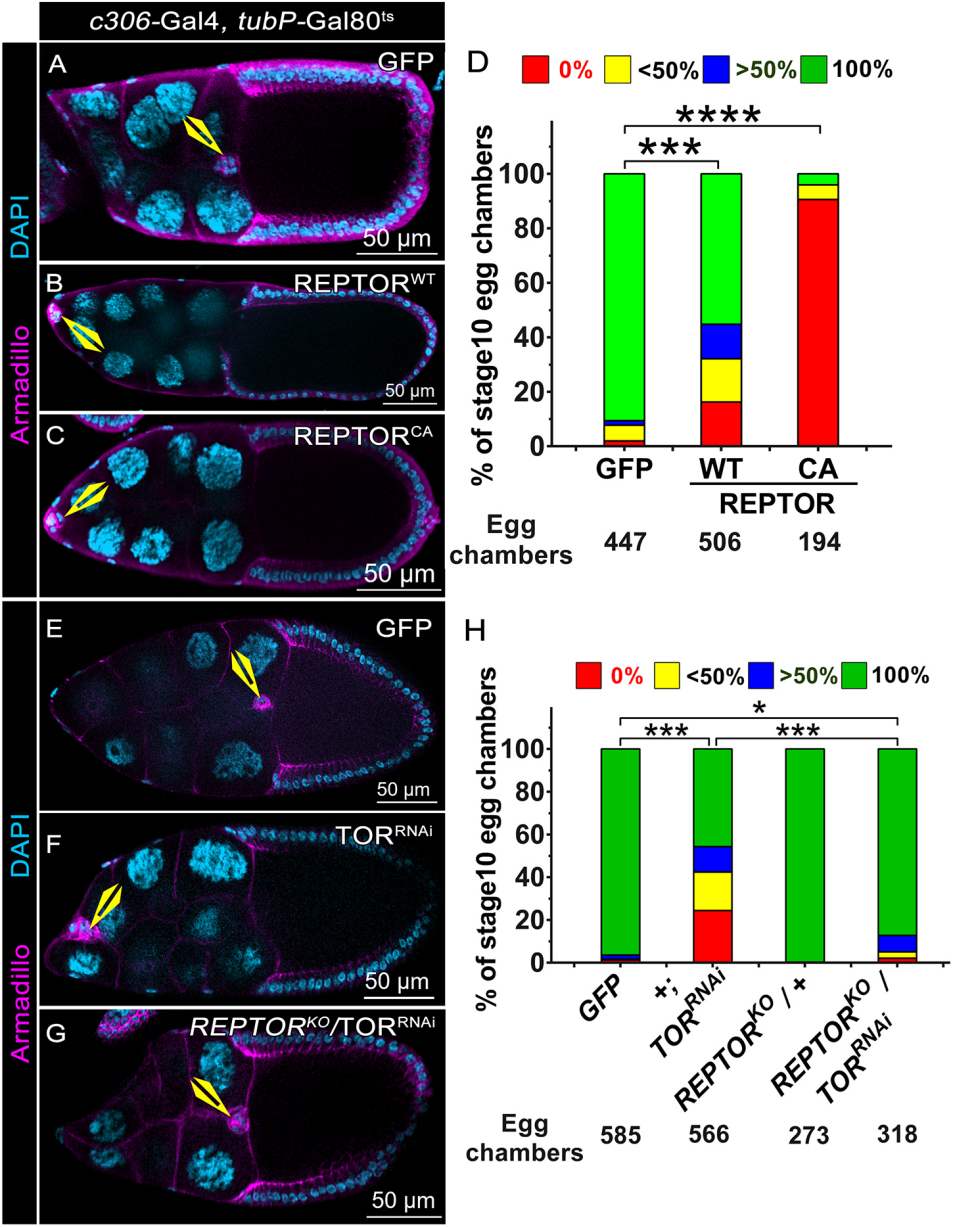
**BC migration is negatively regulated by REPTOR activity.** (A-C,E-G) Stage 10 egg chambers of the indicated genotypes; Armadillo (magenta) and DAPI (cyan). Yellow arrowheads indicate BCs. (D) Quantification of migration efficiency for the egg chambers shown in A-C. (E-H) 50% downregulation of REPTOR activity in TOR-depleted clusters rescues the phenotype. (H) Quantification of migration efficiency for the egg chambers shown in E-G. The colour code in D and H represents the distance covered from the anterior follicle cell. ****P<0.0001, ***P<0.001, *P<0.05 (non-parametric Mann–Whitney test). *N*≥3.

We know TORC1 alters the transcriptional status of a cell by modulating the activity of transcription factor REPTOR (Repressor of TOR). Active TORC1 phosphorylates REPTOR and promotes its cytosolic retention by the 14-3-3 group of proteins (Tiebe et al., 2015). While under conditions of TORC1 inactivity, REPTOR is no longer phosphorylated and hence translocates into the nucleus and modulates transcription along with its partner REPTOR-binding partner (REPTOR-BP). In *Drosophila* S2 cells, REPTOR activity regulates the expression of 90% of genes observed upon TORC1 inhibition (Tiebe et al., 2015). In the context of this information, we were curious to find out whether the ectopic expression of REPTOR is inhibitory to BC migration. To test this, we first overexpressed wild-type (WT) and constitutively active (CA) forms of REPTOR individually in the BCs using a *c306*-GAL4 driver along with *tubP-*Gal80^ts^. We observed an MD of around 45% in REPTOR^WT^ overexpression and 95% defect in REPTOR^CA^ overexpression, compared to 10% in control egg chambers. In addition, approximately 15% and 90% of the clusters failed to delaminate from the epithelia on REPTOR^WT^ and REPTOR^CA^ overexpression, respectively, compared to 2% in the control (Fig. 3A-D). Thus, it appears that higher activity of REPTOR can negatively regulate BC migration and severely impede delamination of the cluster. To further support our finding that REPTOR overexpression is detrimental to BC migration, we investigated whether the BC MD observed upon TOR depletion is indeed due to an increase in REPTOR function. We examined this by genetically decreasing the levels of REPTOR in the TOR-depleted clusters and examining their migration efficiency. Our premise was that if a higher level of REPTOR is the cause of migration and detachment defects observed upon TOR knockdown, then decreasing REPTOR should rescue these defects.

To test this, we employed the milder protocol (refer to Materials and Methods) for TOR knockdown in the presence and absence of the *REPTOR^KO^* null allele (Tiebe et al., 2015). TOR depletion in a WT background (Fig. 3F,H), resulted in 54% MD and 24% detachment defects (DD), while the control exhibited 3.6% MD and 1.3% DD (Fig. 3E,H). Interestingly, removing one copy of the *REPTOR* allele in the TOR-depletion background resulted in the MD and DD dropping to 12.8% and 2.1%, respectively (Fig. 3G,H). Likewise, the DD observed in TOR and REPTOR depletion were comparable to control values, suggesting a strong rescue of detachment defects (Fig. 3H). These results suggest that the BC MD observed in the TOR-depletion background is due to the upregulation of REPTOR function per se.

### Diap1 is a downstream target of TOR in migrating BCs

**Fig. 4.**
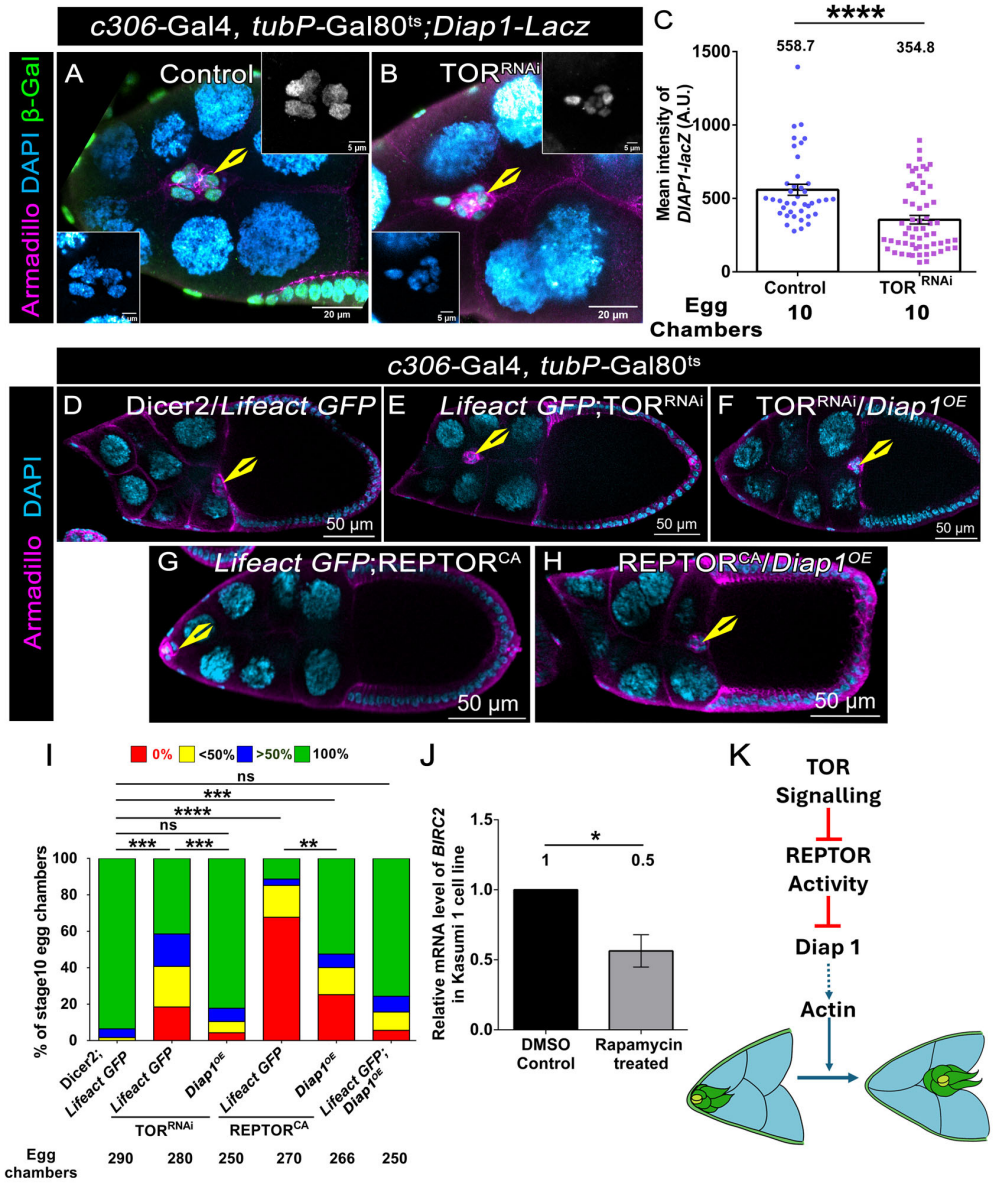
**Diap1 is a downstream target of TOR in migrating BCs.** (A,B,D-H) Egg chambers of the indicated genotypes. Armadillo (magenta), DAPI (cyan). Yellow arrowheads marks BCs. (A,B) Stage 9 egg chambers. *Diap1-lacZ* is in green (right inset in grey). Left inset shows DAPI. (C) Quantification of mean intensity suggests *Diap1*-*lacZ* is lower in TOR-depleted clusters. ***P<0.001 (non-parametric, two-tailed, unpaired Student's *t*-test). *N*≥3. (D-H) Stage 10 egg chambers. (I) Quantification of migration efficiency. The colour code indicates the distance covered from the anterior end ****P<0.0001, ***P<0.001, **P<0.01 (non-parametric Mann–Whitney test). ns, not significant (*P*≥0.5). *N*≥3. (J) Quantification of the relative transcript level of *BIRC2* in rapamycin-treated Kasumi-1 cells. *P<0.05 (non-parametric, two-tailed, unpaired Student's *t*-test). *N*=3. All error bars represent s.e.m. (K) The model for *Drosophila* TOR signalling suggests that TORC1 regulates BC migration by downmodulating REPTOR, which in turn negatively regulates Diap1 and F-actin. Green represents BCs, yellow is for polar cells. Nurse cells are cyan.

To identify the downstream target of TORC1 that modulates efficient cluster movement, we searched the literature for targets of TOR across various systems, spanning both plants and animals. Among all the targets (Table S1) (Goberdhan et al., 2005; Hay and Sonenberg, 2004; Luo et al., 2020; Mukherjee and Duttaroy, 2013), we focused on Death-associated inhibitor of apoptosis 1 (Diap1) (Parker and Struhl, 2015). This was primarily because Diap1 had been shown to regulate the BC movement. Interestingly, Diap1 mutant follicle cells exhibit lower levels of F-actin, similar to what we observed in TOR-depleted clusters (Geisbrecht and Montell, 2004). First, we examined the levels of *Diap1* transcripts in TOR-depleted larvae and found them to be 70% lower than in the control (relative transcript level in TOR^RNAi^=0.3; Fig. S5A,B). To verify whether the observed Diap1 downregulation was indeed mediated by the transcriptional modulator REPTOR, we examined *Diap1* transcript levels in the background of REPTOR^CA^. We observed lower levels of *Diap1* transcripts in the REPTOR^CA^-overexpressing organisms, suggesting that REPTOR modulates Diap1 at the transcriptional level to an extent similar to that observed for TOR (relative transcript level in REPTOR^CA^=0.3; Fig. S5A,B). We also assessed *Diap1* transcription in TOR-depleted BC clusters using the standard reporter *Diap1-lacZ* and observed its levels were significantly reduced compared to the control (mean intensity of *Diap1-lacZ* in control=558.7 A.U.; TOR^RNAi^=354.8 A.U) (Fig. 4A-C) (Koontz et al., 2013; Yu and Pan, 2018). Next, we wanted to check whether the lower level of *Diap1* transcripts observed in the TOR-depletion background was indeed the reason for the observed MD of the BC clusters. To test this, we overexpressed Diap1 in the TOR-depleted clusters and examined their migration efficiency. We observed that Diap1 overexpression partially rescued the MD of the TOR-depleted clusters compared to the control (43.0% MD in UAS *Lifeact GFP*; UAS TOR^RNAi^, 18.12% MD in UAS *Diap1*/UAS TOR^RNAi^; Fig. 4D-F,I). We also observed that Diap1 overexpression partially rescued both detachment and MD exhibited by clusters with hyperactive REPTOR (78.10% MD in UAS *Lifeact GFP*; UAS *REPTOR^CA^*, 46.86% MD in UAS *Diap1*/UAS *REPTOR^CA^*, Fig. 4G-I). Diap1-overexpressing, TOR-depleted clusters exhibited higher levels of F-actin (average intensity in UAS *Lifeact GFP*; UAS TOR^RNAi^ -0.6, in UAS *Diap1*/UAS TOR^RNAi^*-*1.1; Fig. S5D-F) and number of actin fibres compared with that observed in the TOR-depleted clusters (average no of F-actin fibres in UAS *Lifeact GFP*; UAS TOR^RNAi^ -21.9, UAS *Diap1*/UAS TOR^RNAi^*-*45.1; Fig. S5D,E,G), suggesting that Diap1-mediated rescue is dependent on actin. Altogether, our genetic data suggest that TORC1 modulates the levels of Diap1 through REPTOR, facilitating BC detachment and their efficient movement towards the oocyte. Since Diap1 is a TOR target, we were curious about whether this regulatory axis can be translated to higher systems.

### Rapamycin-treated Kasumi-1 cell line exhibits lower BIRC2

The human homologue of Diap1 is baculoviral IAP repeat containing 2 (BIRC2) (Gesellchen et al., 2005). Since BIRC2 inhibits apoptosis like Diap1, we investigated whether TOR might regulate BIRC2 in mammalian cells, similar to how it governs Diap1 in flies. Specifically, we investigated the transcript levels of BIRC2 in TOR-depleted cells. Rapamycin is a protein kinase inhibitor and a specific inhibitor of TORC1. Acute myelogenous leukaemia is a heterogeneous group of malignancies, and we know that the mTOR pathway plays a central role in their survival and proliferation (Barrett et al., 2012; Xu et al., 2003). We employed the myeloblast cell line Kasumi-1, which is sensitive to the drug rapamycin, to test our hypothesis. We treated the Kasumi-1 cells with 20 nM rapamycin for 24 h, extracted the RNA, then carried out qRT-PCR with BIRC2 primers. We found that the level of *BIRC2* transcript was approximately 50% lower in rapamycin-treated Kasumi-1 cells compared to that observed for the DMSO-treated controls (Fig. 4J). In addition, we observed that F-actin levels were reduced in the rapamycin treatment group (relative intensity=1±0.07) compared to DMSO-treated controls (relative intensity=0.2±0.01) (Fig. S6). These results indicate that TOR regulates BIRC2 expression in Kasumi-1 cells. Altogether, our findings identify Diap1 as a target of TORC1 in collectively migrating BCs. Notably, this mode of regulation can be extrapolated to the mammalian system, as the transcripts of *BIRC2* (Diap1 homologue) exhibit susceptibility to rapamycin treatment.

Our data demonstrate that TORC1 functions through Diap1 to modulate F-actin in migrating BCs. Although our results suggest that TOR regulates Diap1 at the transcriptional level, it is unclear whether the effect is direct or indirect. Although we do not understand the reason behind the appearance of more pronounced rearward protrusions in the TOR-depleted cluster, it would be worth examining this aspect concerning the overall polarity of these BC clusters. Nevertheless, Diap1 appears to be a downstream target of TOR in the migrating BCs (Fig. 4K). Our investigation in Kasumi-1 cells documents lower levels of *BIRC2* when treated with rapamycin. This suggests that our experimental findings may be extrapolated to mammalian systems, and it would be worth examining the status of BIRC2 in highly tumorigenic cell lines in which the driving force is misregulated TOR signalling.

## MATERIALS AND METHODS

### *Drosophila* stocks

Fly stocks were maintained at 25°C. *c306-*GAL4, *upd*-Gal4, *hsFLP; FO-P[actin-<17b >Gal4]* and *actin*-Gal4 were employed for overexpression (Montell et al., 1992). The following fly lines were used: UAS TOR RNAi constructs [Bloomington *Drosophila* Stock Center (BDSC) 33951 and BDSC 34639), UAS Raptor RNAi (BDSC 41912), UAS Rictor RNAi (BDSC 36584), UAS Rictor RNAi (BDSC 31388), UAS Rictor RNAi (BDSC 31527), UAS Rictor RNAi (BDSC 36699), UAS PRAS 40 (gifted by Aurelio Teleman; Pallares-Cartes et al., 2012), UAS REPTOR-WT (gifted by Aurelio Teleman; Tiebe et al., 2015), UAS REPTOR CA (gifted by Aurelio Teleman; Tiebe et al., 2015), UAS Lifeact-GAP (BDSC 35544), UAS Diap1 (BDSC 6657), UAS GFP (BDSC 6874), *TOR*^Δ*P*^ loss-of-function mutation (BDSC 7014), *REPTOR^KO^* null allele (gifted by Aurelio Teleman; Tiebe et al., 2015), y1 w*; P{lacW}Diap1j5C8/TM3, Sb1 (BDSC 12093).

For MARCM, *TOR*^Δ*P*^*,FRT 40A* was crossed to *hsFLP, UAS-mCD8GFP; FRT40A, tubP-Gal80* (BDSC 42725/*CyO-TM6Be*). One- to two-day-old F1 flies were collected and incubated at 37°C for 1 h, three times a day with a minimum 2-h interval in between the subsequent heat shocks. Heat shock was given for three consecutive days and flies were fattened at 25°C after 5 days for 20-22 h and then dissected.

To modulate TOR and REPTOR in the BC cluster, we crossed TOR^RNAi^ and REPTOR^WT^, REPTOR^CA^ with *c306*-Gal4, *tubP*-Gal80^ts^. The crosses were set up at 16°C. Two-day-old post-eclosion the flies were fattened at 31°C for 24-30 h, followed by 24-30 h at 29°C in a freshly yeasted vial, and then dissected.

### Immunohistochemistry

For immunostaining, the following primary antibodies were used: mouse anti-Armadillo monoclonal [N27A1, Developmental Studies Hybridoma Bank (DSHB); 1:50], mouse anti-β-Gal (40-1a, DSHB; 1:100), rabbit anti-β-Gal (A-11132, Invitrogen; 1:500), rabbit anti-pS6 (gifted by Teleman laboratory, DKFZ, Heidelberg, Germany; 1:200). Ovaries were dissected in Schneider's S2 media, fixed with 4% paraformaldehyde (PFA) (158127, Sigma-Aldrich) and blocked using PBT solution [5% bovine serum albumin (Amresco, 0332), 0.3% Triton X-100 (Affymetrix, T1001a) in PBS (Sigma-Aldrich, P3813). Resuspended ovaries were then incubated with primary antibody overnight at 4°C. Secondary antibodies goat anti-mouse IgG (H+L) cross-absorbed secondary antibody, Alexa Fluor™ 594 (A11005, Invitrogen), goat anti-mouse IgG (H+L) cross-absorbed secondary antibody, Alexa Fluor™ 488 (A11001, Invitrogen), goat anti-rabbit IgG (H+L) cross-absorbed secondary antibody, Alexa Fluor™ 568 (A11011, Invitrogen), chicken anti-rabbit IgG (H+L) cross-absorbed secondary antibody, Alexa Fluor™ 488 (A21441, Invitrogen), were used at a dilution of 1:400.

For phalloidin staining, the ovaries were dissected, and each ovariole was pulled out of the muscular sheath very carefully. The ovarioles were fixed with 4% PFA for 15 min at room temperature and rinsed with PT (1× PBS with 0.3% Triton X-100). The egg chambers were incubated with phalloidin (Invitrogen; 1:500) diluted with 0.3% PT for 15 min at room temperature, followed by normal staining protocol (Montell et al., 1992).

### Cell culture and rapamycin treatment

The Kasumi-1 acute myeloid leukaemia cells were obtained from ATCC (CRL-2724; RRID: CVCL_0589). The cells were cultured in RPMI 1640 (Thermo Fisher Scientific, A1049101) supplemented with 20% fetal bovine serum (Thermo Fisher Scientific, 10270-106), 100 µg/ml of Pen Strep (Thermo Fisher Scientific, 15140-122). Cells were maintained at 37°C in a humidified CO_2_ incubator (5% CO_2_). Twelve hours before treatment, cells were seeded into 6-well plate at a density of 1-2×10^6^ cells per well. Cells were treated with 20 nM rapamycin for 24 h. (Altman et al., 2014).

### qRT-PCR

Total RNA samples were isolated from cells using the RNeasy Mini Kit (QIAGEN, 74104), according to the supplier protocol. Forward and reverse primers of target genes were designed using Primer 3 software. cDNA was synthesised using the Thermo Fisher Scientific cDNA synthesis kit (K1622) qRT-PCR was performed on RNA samples using PowerSYBR Green PCR Master Mix (Applied Biosystems, 4367659) in a 384-well plate. (10 min at 95°C, 40 cycles of 15 s at 95°C, 30 s at 58°C and 30 s at 72°C). Finally, melting was carried out at 60-95°C (at an increment of 1.6°C per step and a holding time of 5 s for each step). For carrying out qRT-PCR, 0.5 µl of 10 mM concentration of *GAPDH* and *BIRC2* primers were added to 10 µl reaction mixture: *GAPDH* forward sequence, 5′-GGAAGGTGAAGGTCGGAGTC-3′; *GAPDH* reverse sequence, 5′-TGAGGTCAATGAAGGGGTCA-3′; *BIRC2* forward sequence, 5′-TGGTTAAAGGAAATGCTGCGG-3′; *BIRC2* reverse sequence, 5′-GCATACTACCAGATGACCACAAG-3′. *GAPDH* was used as a housekeeping control.

### RT-PCR

RNA isolation was carried out on the ovaries of adult flies using TRIzol reagent followed by cDNA preparation. The status of the *Diap1* and *rictor* transcripts was evaluated by RT-PCR. *Rp49* (*RpL32*) forward sequence, 5′-CTAAGCTGTCGCACAAATGGC-3′; *Rp49* reverse sequence, 5′-AACTTCTTGAATCCGGTGGGC-3′; *Diap1* forward sequence, 5′-TCAGAGGAAGAGCAGCAGAC-3′; *Diap1* reverse sequence, 5′-ATATACGCGCATCACATCGG-3′; *rictor* forward sequence, 5′-GCGTCACCTCCATAACCCG-3′; *rictor* reverse sequence, 5′-ACCGCAGATGTTCCTCGTTTG-3′. *Rp49* was used as a housekeeping control.

### Live imaging and analysis

Flies used for live-cell imaging of BC migration were of the genotype *c306*-GAL4, *tubP*-Gal80^ts^; UAS *Lifeact-GFP*/UAS *dsRed* (control) and *c306*-GAL4, *tubP*-Gal80^ts^; UAS *Lifeact-GFP*; UAS TOR^RNAi^ (experiment). This unique combination of RFP as control and the absence of RFP in the experimental genotype allowed us to perform simultaneous live imaging of both control and TOR-depleted BCs under identical culture conditions. For live imaging, the following setup was used: Olympus IX81 microscope body, CoolSnap Myo camera, PRIOR motorised stage and software interface through open-source Micromanager. Time-lapse microscopy was performed as described by Prasad et al. (2007). Frames were taken at 2 min intervals. Culture conditions were kept same for both control and experiments. On average each movie lasted for around 3-4 h.

### Data quantitation and statistics

#### Protrusion analysis

Cluster velocity and number, orientation, length and stability of protrusions were measured manually (Felix et al., 2015). Velocity was calculated only for the duration of the cluster migration for detached clusters. To study protrusion dynamics the number, length and stability of protrusions were evaluated. To measure the length of the protrusion, a line was drawn from the centre of the cluster to the extension tip. The length of this line was measured using ImageJ software. Any extension longer than the radius of the cluster was taken as protrusion (approximately, extensions greater than 12 pixels were considered as protrusions). To infer the orientation of this protrusion, the angle of this line with respect to the *x*-axis in a two-dimensional plane was calculated. Protrusions were classified as front (0°-45° and 0°-315°), side (225°-315° and 45°-135°) and back (135°-225°). The duration (number of frames) for which the protrusion lasted was considered for evaluating its stability. Since the total number of protrusions varied between BC clusters, we normalised the data by considering the total number of protrusions in each cluster as 100%. We then quantified the percentage of protrusions distributed on each side of the cluster. The mean values of these percentages were plotted as a pie chart. For statistical analysis, we performed a one-way ANOVA test and a two-tailed, nonparametric Mann–Whitney test.

#### Quantification of efficiency of BC migration

Only stage 10 egg chambers were considered for scoring MD. Stage 10 egg chambers can be identified by observing the size of the oocyte, which occupies half of the total volume of the egg chamber. The total path of the BC cluster (from the anterior end to the oocyte boundary) was divided into four parts. Based on the distance covered by the BC cluster at stage 10, the egg chamber was placed under any of these categories: 0%/undetached defect in migration, <50% migration, >50% migration, 100% migration. The total number of BC clusters belonging to the individual category was counted, and the percentage MD was calculated was using the following formula:

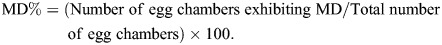


#### Actin fibre quantification

*z*-stack images of migratory BC clusters were acquired at regular *z*-intervals (300 nm) using an ABBERIOR 3D STED facility line microscope with a 60× magnification objective. Imaging was performed using 25% depletion laser power, with accumulation set to 3, dual time set to 5, a pixel size of 30 nm (*xy*), and a pinhole size of 1 Airy Unit (AU). At least five BC clusters were analysed per condition (control and experimental) to quantify the number of actin fibres and mean phalloidin intensity.

The acquired *z*-sections were processed using ImageJ software. A scale bar was calibrated using reference images taken under the same imaging settings, and the pixel-to-micrometre conversion was applied based on the 60× magnification. Cytoplasmic actin fibres within the BC clusters were manually traced using the Freehand selection tool, and fibre lengths were measured using the ‘Measure’ function in ImageJ. The fibres were analysed by examining each *z*-plane of the cluster. In the control, the minimum length of actin fibre was set as 1 µm as this was the smallest length encountered in the migrating BC clusters. Thus, for our analysis, only fibres with a length greater than 1 µm were considered. A bar diagram was generated using GraphPad Prism (version 9.5.0) in which the individual data points represent the number of actin fibres of each BC cluster.

To assess actin, immunostaining was performed using rhodamine phalloidin. The BC clusters from egg chambers were imaged at 100× magnification, at regular *z*-intervals using an inverted Ti2 eclipse microscope (Nikon, Japan) with confocal module (Nikon AX model, with NIS elements software) with minimal crosstalk, employing sequential Galvano scanning and a 1 AU pinhole capturing *z*-sections at regular 300 nm intervals with consistent laser power for both experimental and control samples. The *z*-stack images were merged to generate a 2D maximum intensity projection (MIP) image. The individual BC nucleus was outlined in the MIP image, and the mean phalloidin intensity was quantified using ImageJ software. The values were then normalised to the mean DAPI intensity of the nuclei of corresponding BC. The average mean intensity was plotted.

#### *Diap1-lacZ* intensity quantification

*Diap1-lacZ* contains a regulatory sequence from the *Diap1* gene that drives the expression of the *lacZ* reporter gene, reflecting the transcriptional activity of the *Diap1* locus (Wu et al., 2003). However, this construct does not provide information on the translation or production of Diap1 protein.

To assess *lacZ* levels, immunostaining was performed using a primary antibody against the β-Gal protein. The BC clusters from egg chambers were imaged at 100× magnification, at regular *z-*intervals using an inverted Ti2 eclipse microscope (Nikon, Japan) with confocal module (Nikon AX model, with NIS elements software) with minimal crosstalk, employing sequential Galvano scanning and a 1 AU pinhole capturing *z*-sections at regular intervals with consistent laser power for both experimental and control samples. The *z-*stack images were merged to generate a 2D MIP image. The individual BC nucleus was outlined in the MIP image, and the mean *lacZ* intensity was quantified using ImageJ software. The values were then normalised to the background intensity of the corresponding sample. The average mean intensity was plotted.

#### Phalloidin intensity quantification

Kasumi-1 cells were adhered to poly-L-lysine-coated coverslips this was followed by fixation with 4% PFA, permeabilisation with 0.1% PBST (1× PBS with 0.1% Triton X-100) and two washes with 1× PBS, blocking with 3% bovine serum albumin followed by phalloidin (49409, Sigma-Aldrich; 1:200) staining and mounting. The cells were imaged at 100× magnification, at regular *z*-intervals using an inverted Ti2 eclipse microscope (Nikon, Japan) with confocal module (Nikon AX model, with NIS elements software) with minimal crosstalk, employing sequential Galvano scanning and a 1 AU pinhole. The *z*-stack images were merged to generate a 2D MIP image. The individual cells were outlined in the MIP image, and the mean phalloidin intensity was quantified using ImageJ software. The values were then normalised to the DAPI intensity of the corresponding sample. The average intensity of the control samples was set to 1, and the experimental intensities were expressed as fold changes relative to the control.

#### Statistical analysis

Difference of means were determined by two-tailed, unpaired *t*-test, Mann–Whitney test and one-way ANOVA (nonparametric) test of unequal variance in GraphPad Prism 9.5.0. All error bars indicate s.e.m. All figures utilise the following range for assigning significance of means: ****P<0.0001, ***P<0.001, **P<0.01, *0.05<*P*<0.01 and *P*>0.05 as not significant.

## Supplementary Material

10.1242/develop.204612_sup1 Supplementary information
