# Supplementary material for: TOR signalling mediates the collective movement of border cells in Drosophila oogenesis
Source: Development. 2025 Sep 4;152(17):dev204612. doi: 10.1242/dev.204612 (PMC12448311; doi:10.1242/dev.204612)
Supplement: Supplementary information [file develop-152-204612-s1.pdf]

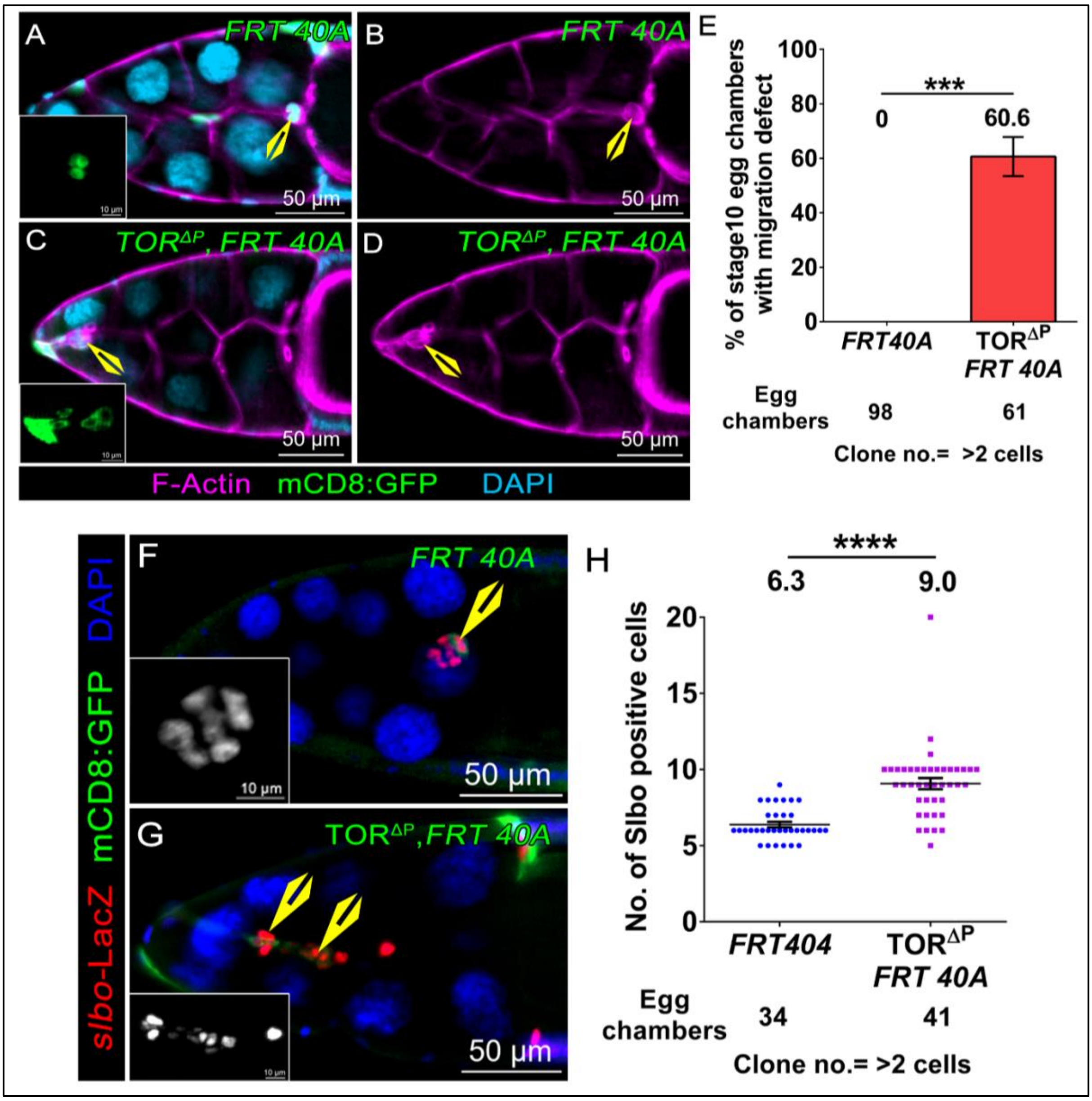

Fig. S1. MARCM analysis of TOR mutant in border cell migration:

(A-E) MARCM analysis. **(A-B)** Control *FRT 40A* mosaic egg chambers stained for F-Actin (Magenta) with DAPI (Cyan), exhibiting complete BCC migration. GFP (Green) marks the clone. White box inset indicates the clone of BCC. **(C-D)** *TOR$^{\Delta P}$,FRT 40A* mosaic mutant egg chambers stained for F-Actin (Magenta) with DAPI (Cyan), exhibiting a BCC detachment defect. GFP (Green) marks the clone of *TOR$^{\Delta P}$* homozygous mutant cells. White box inset indicates the clone of BCC. **(E)** Quantification of migration defect. Error bars indicate SEM.; ****P<0.001*, N=3.

(F-H) Number of Slbo positive cells were high in *TOR$^{\Delta P}$* mutant cluster. **(F)** Control *FRT 40A* mosaic egg chambers stained for *slbo-Lacz* (Red) and with DAPI (Blue, inset Gray). GFP (Green) marks the clone. Yellow arrowheads indicate BCC and White box inset indicates Slbo positive border cells. **(G)** *TOR$^{\Delta P}$, FRT 40A*mosaic mutant egg chambers stained for *slbo-lacz* (Red) and with DAPI (Blue, inset Gray). GFP (Green) marks the clone of *TOR$^{\Delta P}$* homozygous mutant cells. Yellow arrowheads indicate BCC and White box inset indicates Slbo positive border cells. **(H)** Quantification of no. of Slbo positive cells. Error bars indicate SEM. *****P<0.0001,* N=3.

Development • Supplementary information

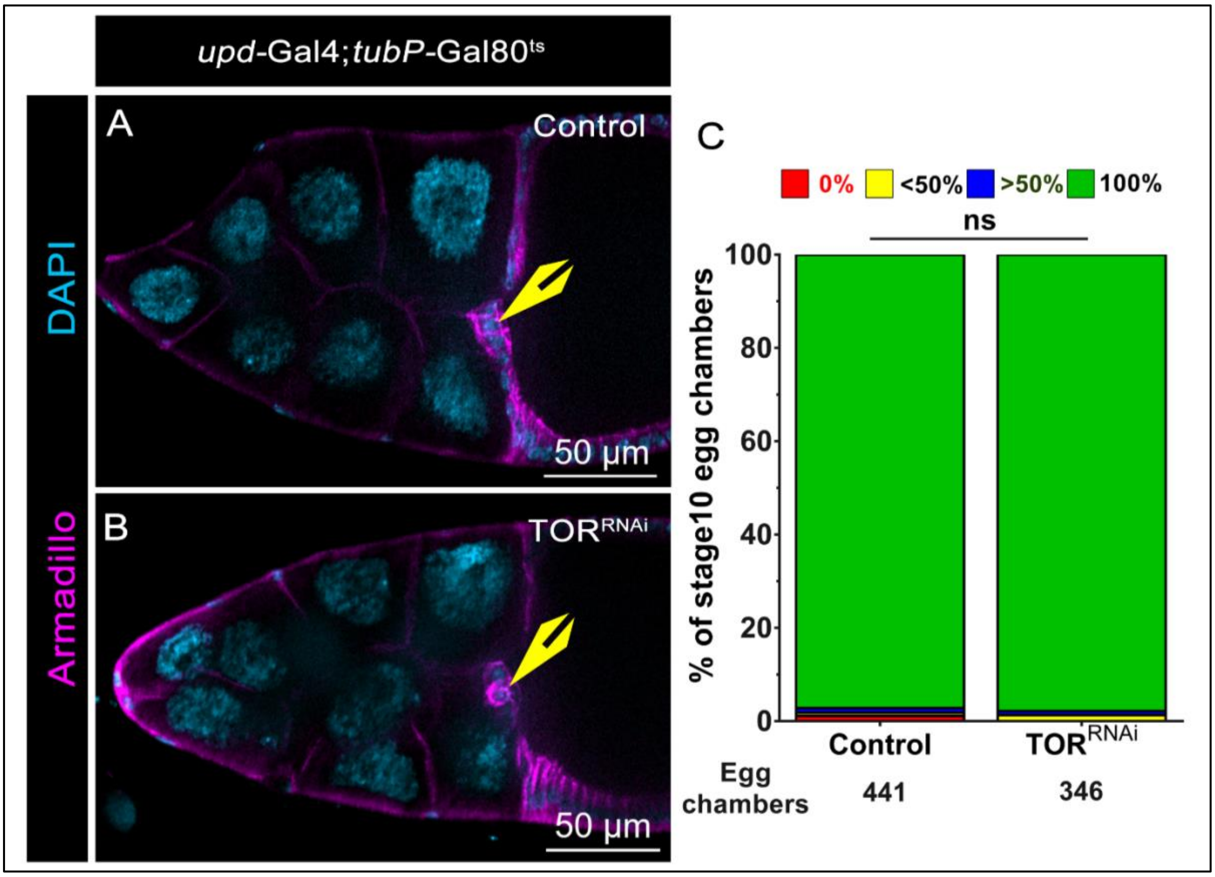

Fig. S2. Downregulation TOR signalling in the polar cell does not affect border cell migration: (A-C) TOR downregulation in polar cell does not affect BC migration. **(A-B)** Stage 10 egg chamber of indicated genotypes, Armadillo (Magenta) and DAPI (Cyan). Yellow arrowheads indicate BCCs. **(C)** Quantification of migration efficiency. The color code indicates the distance covered from the AF cell, ns represents *P=>0.5*, N=3.

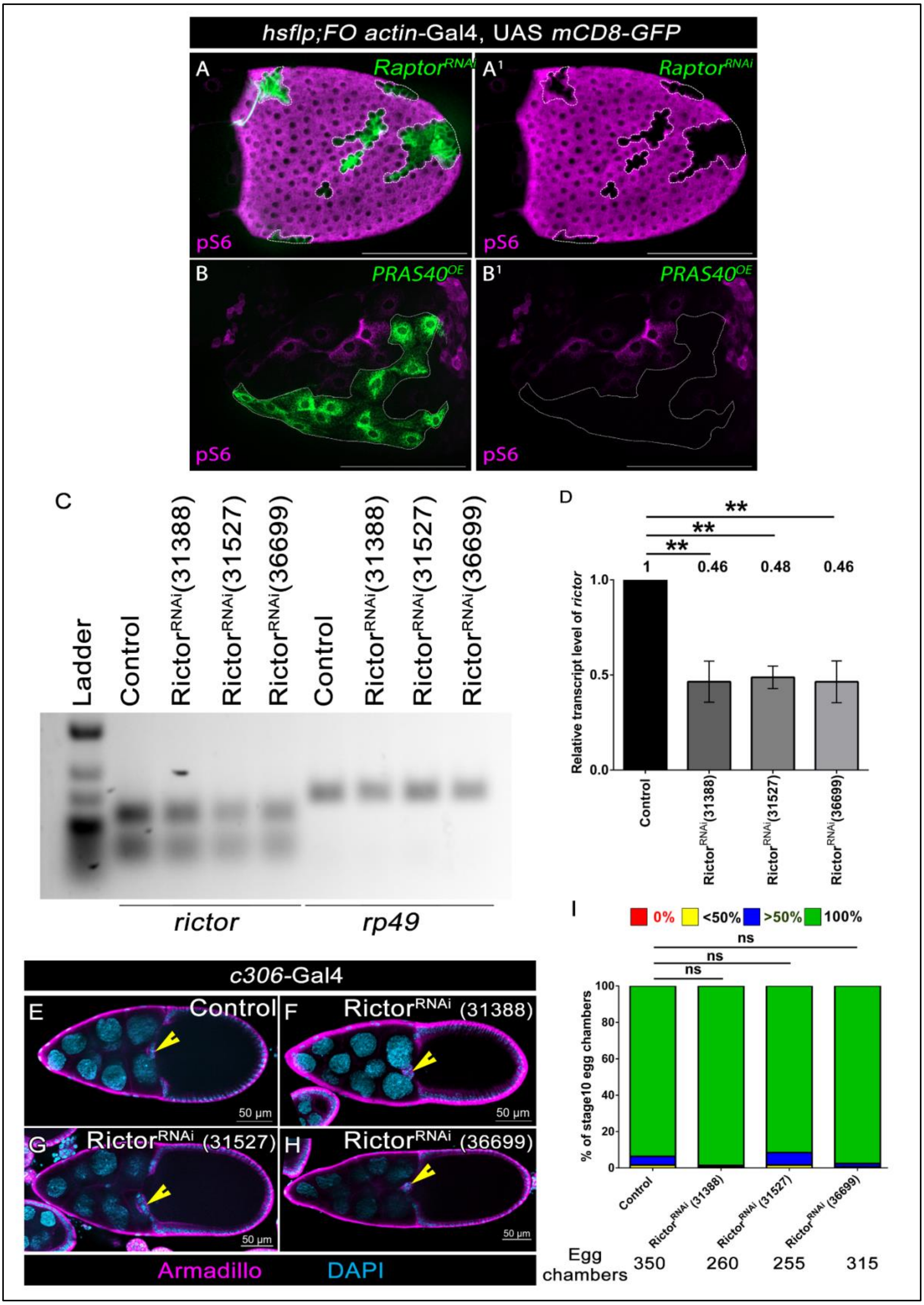
hsflp;FO actin-Gal4, UAS mCD8-GFP
A
Raptor^RNAi
A1
pS6
B
PRAS40^OE
B1
C
Ladder
Control
Rictor^RNAi(31388)
Rictor^RNAi(31527)
Rictor^RNAi(36699)
rictor
rp49
D
**
1
0.46
0.48
0.46
Relative transcript level of rictor
c306-Gal4
E Control
F Rictor^RNAi (31388)
G Rictor^RNAi (31527)
H Rictor^RNAi (36699)
50 µm
Armadillo
DAPI
I
0%
<50%
>50%
100%
ns
% of stage10 egg chambers
Egg chambers
350
260
255
315

Fig. S3. Validation of constructs

(A-A^1) Validation of Raptor RNAi (mCD8GFP, green) by pS6 antibody (magenta). **(B-B^1)** Validation of PRAS 40 overexpression (mCD8GFP, green) by pS6 antibody (magenta). **(C-D)** Validation of Rictor^{RNAi} construct using semi-quantitative RT-PCR, target gene: *rictor* normalised against *rp49.* Error bars denote SEM. ** Represent p-value <0.01 **(E-I)** Rictor downregulation in the border cell does not impede BC migration. **(E-H)** Stage 10 egg chamber of indicated genotypes, Armadillo (Magenta) and DAPI (Cyan). Yellow arrowheads indicate BCCs. **(I)** Quantification of migration efficiency. The color code indicates the distance covered from the AF cell, ns represents *P=>0.5*, N=3.

Development • Supplementary information

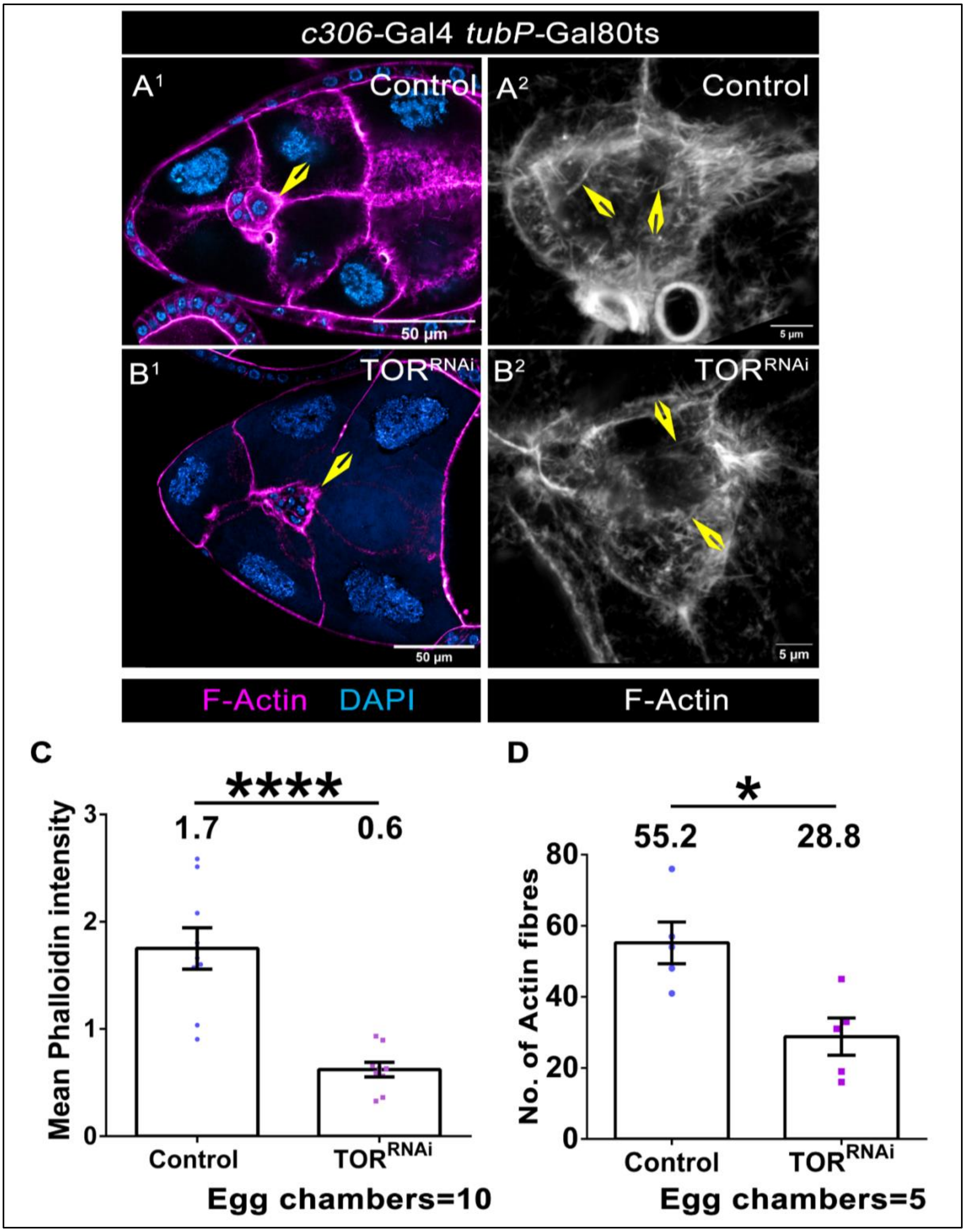

Fig. S4. TOR signaling regulates F-Actin distribution in migratory border cell cluster: **(A1-D)** F-Actin distribution is altered in TOR depleted BCC**.** **(A1,B1)** Egg chambers of indicated genotypes, F-Actin (Magenta) and DAPI (Blue). Yellow arrowheads indicate BCCs. **(A2,B2)** Magnified border cells clusters of indicated genotypes, F-Actin (Gray) and yellow arrowheads indicating Actin fibers**. (C)** Quantification of the mean phalloidin intensity which is normalized with mean DAPI intensity. Error bars represent SEM. ****$P<0.0001$. **(D** Quantification of the Actin fibers numbers in the cluster. Error bars represent SEM. *$P<0.05$.

Development • Supplementary information

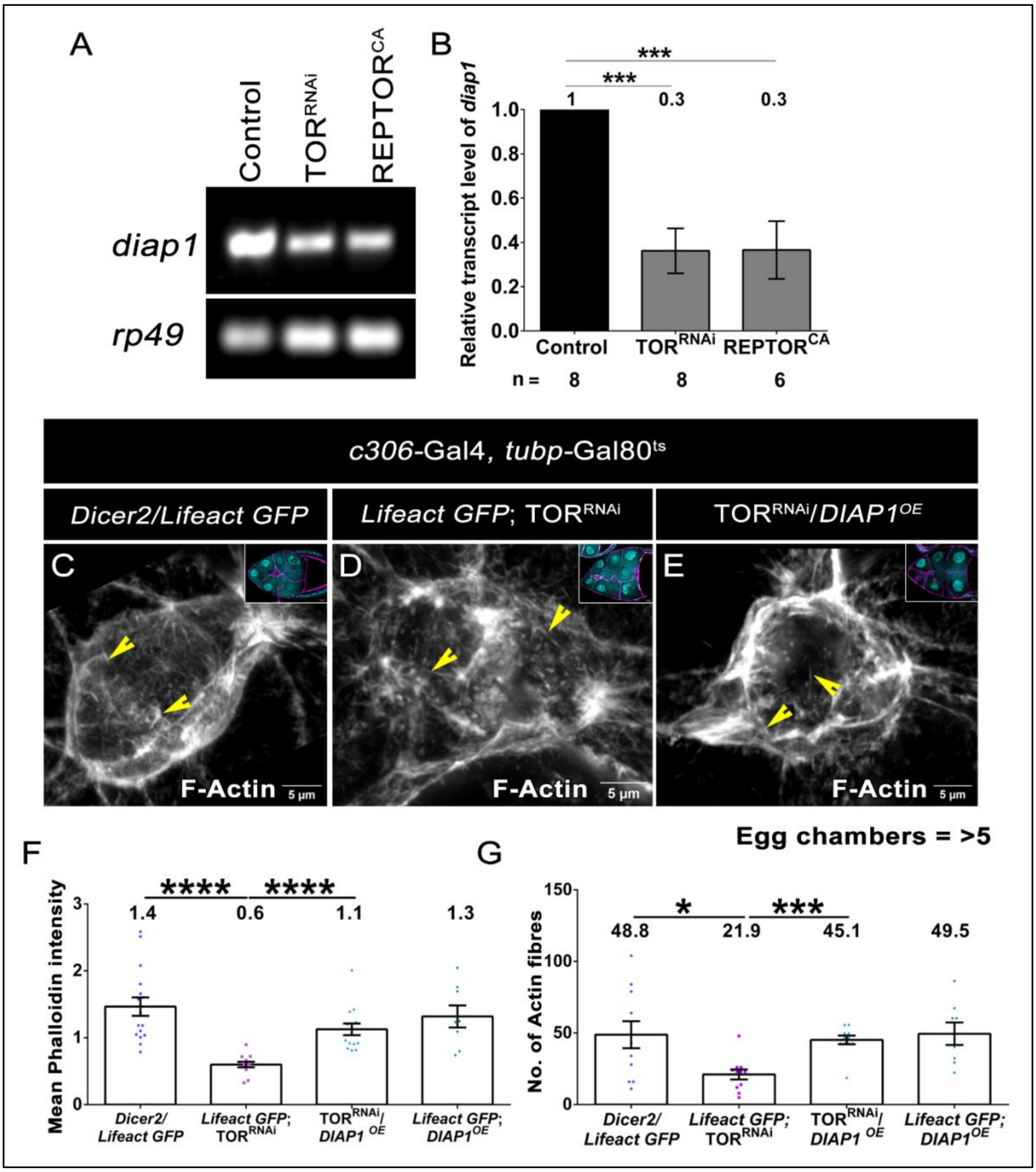

Fig. S5. Overexpression of DIAP1 does not rescue the Actin fibers length:

(A-B) *DIAP1* transcript level altered in TOR^{RNAi} and REPTOR^{CA} larvae. **(A)** Gel image represents the bands of representative gene transcript of indicated genotypes. **(B)** Quantification of Relative transcript level of *DIAP1*. Error bars represent SEM. ****P<0.001*.

(C-G) F-Actin distribution is altered in TOR depleted BCC**. (C-E)** Magnified border cells clusters of indicated genotypes (inset represents the reference egg chambers), F-Actin (Gray) and yellow arrowheads are indicating Actin fibers**. (F)** Quantification of the mean phalloidin intensity which is normalized with mean DAPI intensity. Error bars represent SEM. *****P<0.0001*. **(G)** Quantification of the Actin fibers numbers in the cluster. Error bars represent SEM. ****P<0.001, *P<0.05*.

Development • Supplementary information

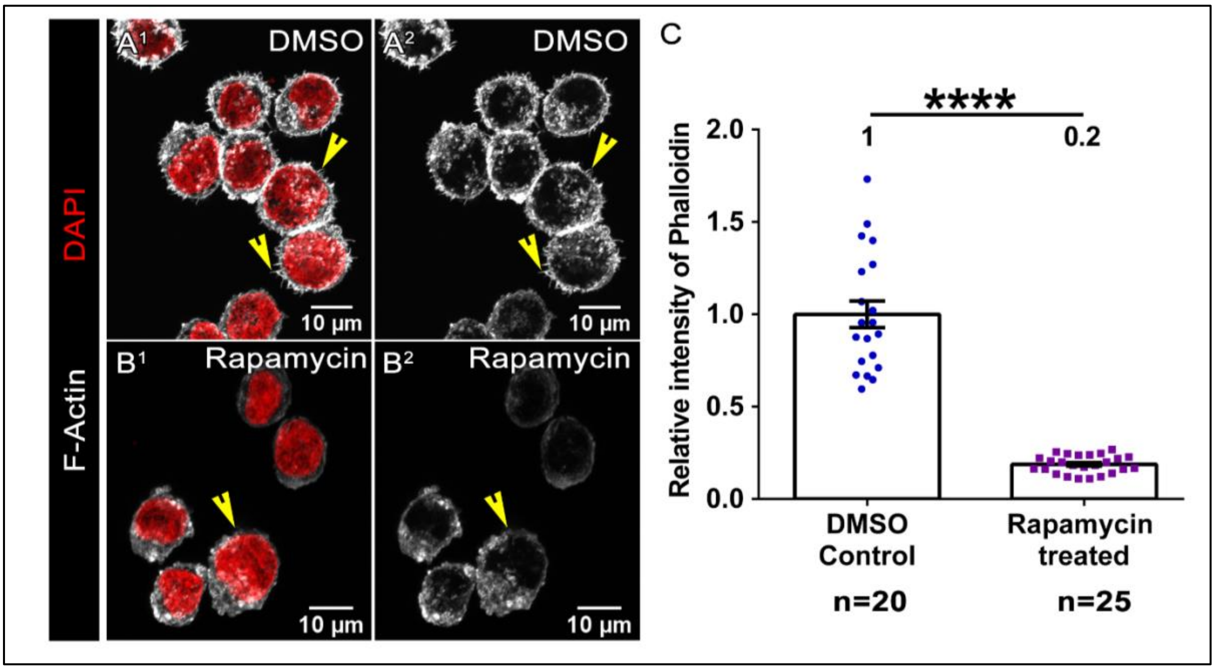

Fig. S6. F-actin is lower in the Rapamycin-treated Kasumi 1 cells:

(A^1-C) Phalloidin intensity was impede in rapamycin treated Kasumi 1 cell line**.** **(A^1-B^2)** Representative images of Kasumi 1 cell lines of indicating treatment conditions, where grey represents F-Actin and red is DAPI, yellow arrowheads indicate Kasumi 1 cells**.** **(F)** Quantification relative intensity of phalloidin. Error bars represent SEM. ****$P<0.0001$, n represents the total no. of cells.

Development • Supplementary information

Table S1. Downstream Targets of TOR signalling

SL No.	Gene name
1	*S6K-1*
2	*4E-BP 1*
3	*eIF4B*
4	*yorkie*
5	*DIAP 1*
6	*four-jointed*
7	*srl*
8	*dSREBP*
9	*Jhl-21*
10	*pathetic*
11	*GDH 2*
12	*dMyc*
13	*TIF1A*
14	*USB*
15	*cyclin D1*

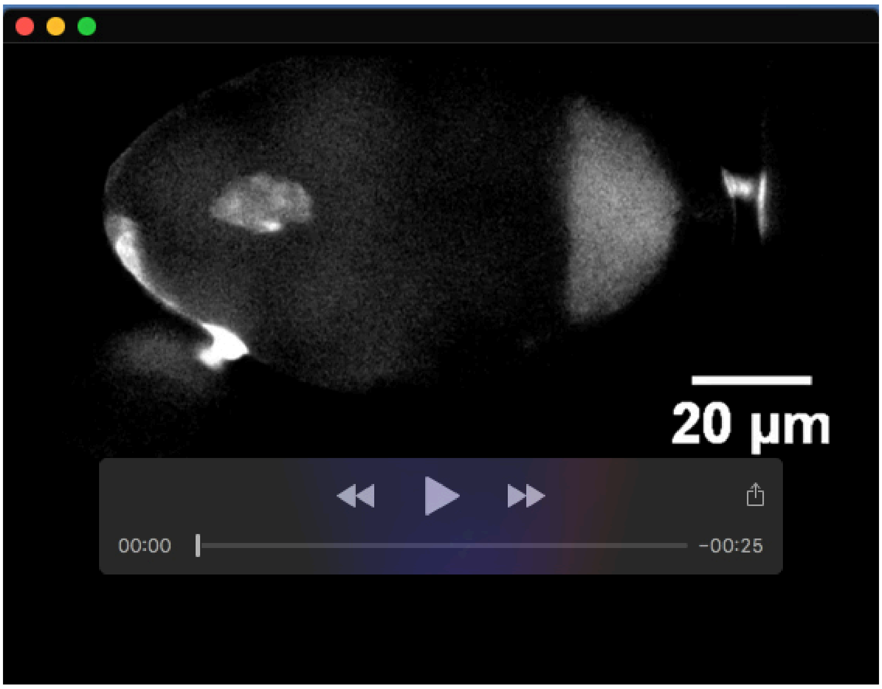

Movie 1. **Time-lapse movie of Stage 9 *Drosophila* egg chamber (Control: *c306*-Gal4, *tubP*-Gal80^{ts}; UAS-*Lifeact-GFP* / UAS-*dsRED-NLS*) captured at 2-minute intervals over a 4-hour period.** The anterior end of egg chamber is towards the left. Lifeact-GFP is expressed specifically in the migrating border cells and labels the cellular protrusion. The border cells exhibit polarized protrusive behavior with long, stable forward directed protrusion from the cluster. Scale bar is shown at the bottom right. The movie is displayed at 10 frames per second (FPS) frame.

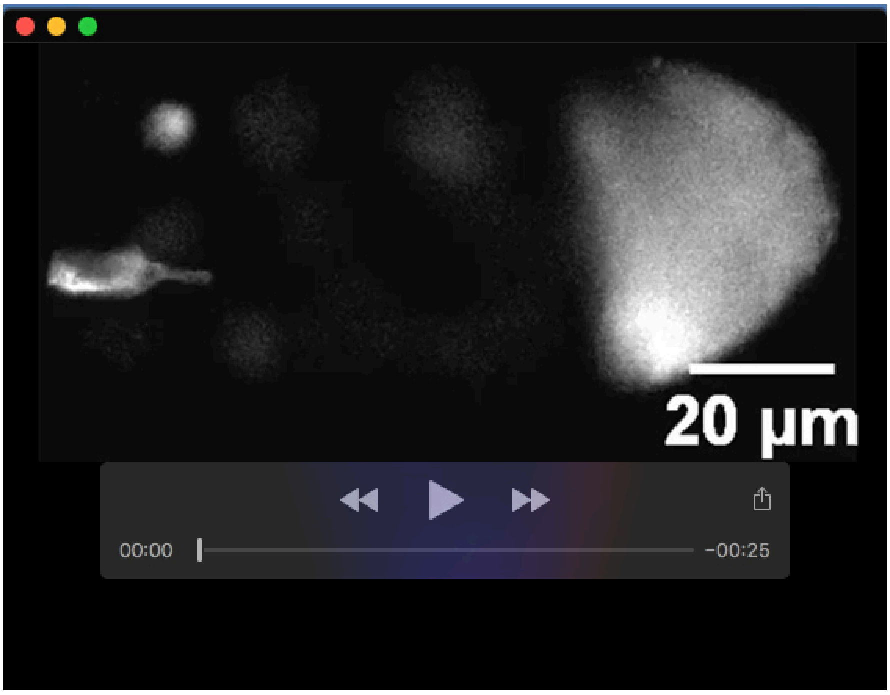

Movie 2. **Time-lapse movie of Stage 9 *Drosophila* egg chamber (Experiment: *c306*-Gal4, *tubP*-Gal80^{ts}; UAS-*Lifeact-GFP*; UAS-TOR^{RNAi}) captured at 2-minute intervals over a 4-hour period.** The anterior end of egg chamber is towards the left. Lifeact-GFP is expressed specifically in the migrating border cells and labels the cellular protrusion. TOR-depleted cluster movement is sluggish, with border cells exhibiting short, misdirected protrusions. Scale bar is shown at the bottom right. The movie is displayed at 10 frames per second (FPS) frame.

Development • Supplementary information

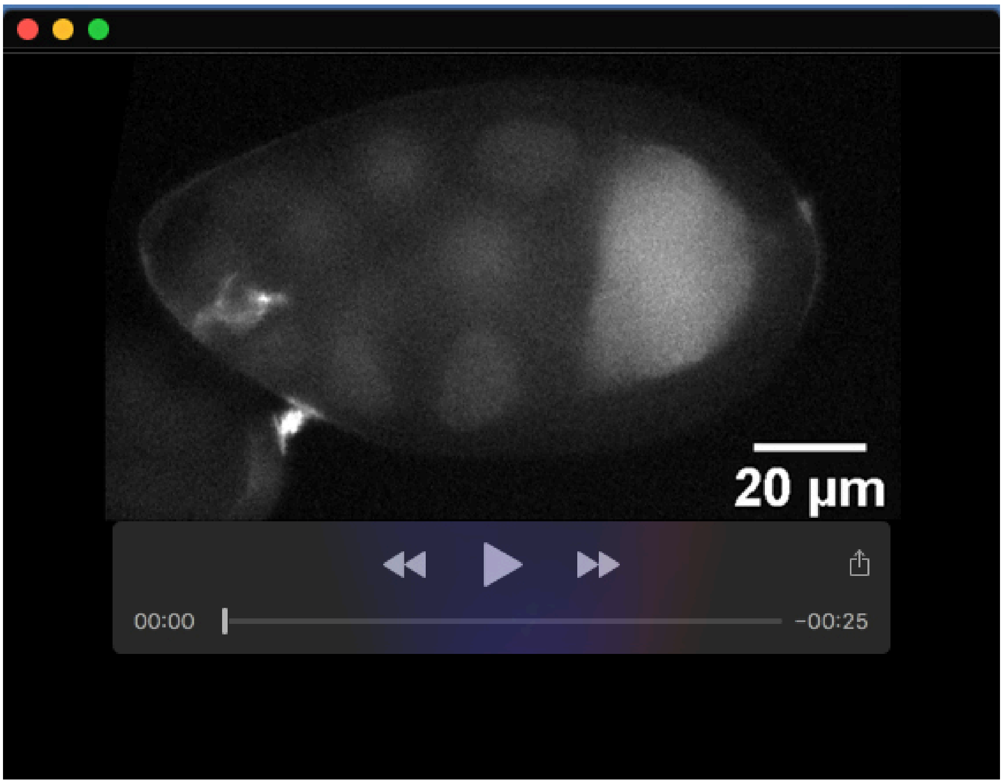

Movie 3. Time-lapse movie of Stage 9 *Drosophila* egg chamber (Experiment: *c306*-Gal4, *tubP*-Gal80^{ts}; UAS-*Lifeact-GFP*; UAS-TOR^{RNAi}) captured at 2-minute intervals over a 4-hour period. The anterior end of egg chamber is towards the left. Lifeact-GFP is expressed specifically in the migrating border cells and labels the cellular protrusion. TOR-depleted cluster movement is sluggish, with border cells exhibiting pronounced short, rearward protrusions in the later part of acquisition. Scale bar is shown at the bottom right. The movie is displayed at 10 frames per second (FPS) frame.
